# Disruption of Glioblastoma Multiforme Cell Circuits with Cinnamaldehyde Highlights Potential Targets with Implications for Novel Therapeutic Strategies

**DOI:** 10.3390/cells12091277

**Published:** 2023-04-28

**Authors:** Shraddha Srivastava, Ketki Patil, Elizabeth W. Thompson, Shadi A. Nakhai, Yoo Na Kim, Casey Haynes, Crystal Bryant, S. Balakrishna Pai

**Affiliations:** Wallace H. Coulter Department of Biomedical Engineering, Georgia Institute of Technology and Emory University, 313 Ferst Drive, Atlanta, GA 30332, USA

**Keywords:** cinnamaldehyde, glioblastoma, apoptosis, ROS, pyruvate kinase (PKM2), phosphomevalonate kinase, U87, proteomics

## Abstract

Glioblastoma multiforme (GBM) is a major aggressive primary brain tumor with dismal survival outcome and few therapeutic options. Although Temozolomide (TMZ) is a part of the standard therapy, over time, it can cause DNA damage leading to deleterious effects, necessitating the discovery of drugs with minimal side effects. To this end, we investigated the effect of cinnamaldehyde (CA), a highly purified, single ingredient from cinnamon, on the GBM cell lines U87 and U251 and the neuroglioma cell line H4. On observing similar impact on the viability in all the three cell lines, detailed studies were conducted with CA and its isomer/analog, trans-CA (TCA), and methoxy-CA (MCA) on U87 cells. The compounds exhibited equal potency when assessed at the cellular level in inhibiting U87 cells as well as at the molecular level, resulting in an increase in reactive oxygen species (ROS) and an increase in the apoptotic and multicaspase cell populations. To further characterize the key entities, protein profiling was performed with CA. The studies revealed differential regulation of entities that could be key to glioblastoma cell circuits such as downregulation of pyruvate kinase-PKM2, the key enzyme of the glycolytic pathway that is central to the Warburg effect. This allows for monitoring the levels of PKM2 after therapy using recently developed noninvasive technology employing PET [^18^F] DASA-23. Additionally, the observation of downregulation of phosphomevalonate kinase is significant as the brain tumor initiating cells (BTIC) are maintained by the metabolism occurring via the mevalonate pathway. Results from the current study, if translated in vivo, could provide additional efficacious treatment options for glioblastoma with minimal side effects.

## 1. Introduction

The majority of malignant tumors of the brain are gliomas. Glioblastoma is an aggressive, major primary brain cancer with poor survival outcome [1]. Glioblastoma multiforme (GBM) is a grade IV tumor that has the ability to proliferate rapidly and become invasive and spreads throughout the brain [2]. The current treatment protocol for GBM is surgical resection, radiation therapy and adjuvant chemotherapy. Being recalcitrant to all the current modalities, GBM patients’ survival rates are dismal at an average of 12–16 months [3]. Many studies are directed towards finding better treatment options, including employing bioengineering strategies such as the use of polymeric nanofibers in animal model systems [4]. Further, better understanding of the heterogeneous nature of GBM and effective treatment options for this cancer are needed. Understanding the cell circuits of GBM better could lead to novel treatment options for this aggressive cancer. Aerobic glycolysis, known as the Warburg effect, is utilized as an energy source by proliferating tumor cells. Furthermore, the mitochondria play a pivotal role as they control key cellular pathways including apoptosis leading to cell death. Thus, targeting mitochondrial function is considered as an important strategy to combat aggressive brain cancers such as GBM [5].

The chemotherapeutic drug currently in the clinic for treating GBM is temozolomide (TMZ), which has improved patient survival and is a DNA methylating agent with slow disease progression when compared to other drugs [6,7]. However, TMZ is reported to cause mutations as observed in the DNA in recurrent glioma [8]. To overcome the long-term deleterious side effects that could occur with TMZ, as well as to treat the glioblastomas that are insensitive to this drug, there is immediate need to discover safer drugs that could be used to efficaciously combat brain tumors. To this end, we opted to study the effect of a purified, single entity namely cinnamaldehyde (CA) (a component of cinnamon) on brain glioma cells (glioblastoma cells U87 and U251 and neuroglioma cells H4) with the anticipation of fewer side effects. Further, we investigated the impact of its isomer/analog TCA and MCA on U87 glioblastoma cell circuits. Importantly, to investigate the impact of CA on glioblastoma U87 cell circuits and elucidate its mechanism of action, we performed multilevel analysis (cellular, molecular and protein profiling) with the aim of identifying potential target molecules to efficaciously combat a devastating tumor such as GBM. 

## 2. Materials and Methods

### 2.1. Cell Lines and Materials

U87, U251 and H4 cell lines were obtained from ATCC (Manassas, VA, USA). The U87 and H4 cells were grown in Dulbecco’s Minimal Essential Medium (DMEM) (Corning, Manassas, VA, USA) supplemented with 10% fetal bovine serum, 1% Penicillin-Streptomycin and 2 mM L-glutamine. U251 cells were cultured in Eagle’s Minimal Essential Medium (EMEM) (Corning, Manassas, VA, USA) supplemented with 10% fetal bovine serum, 1% Penicillin-Streptomycin and 2 mM L-glutamine. Cultures were incubated at 37 °C in an atmosphere of 5% CO_2_. U87 cells expressing enhanced green fluorescent protein (U87eGFP) was derived as reported previously [4] and were used in the present study. Cinnamaldehyde (CA) (cat# W228613), trans-Cinnamaldehyde (TCA) (cat# 239968) and 2-Methoxycinnamaldehyde (MCA) (cat# W318101) were purchased from Sigma Aldrich (St. Louis, MO, USA). A stock solution of concentration of 100 mM of each compound was made in dimethyl sulfoxide. For flow cytometry, a Muse Cell analyzer and Muse assay kits were purchased from Luminex Corporation (Austin, TX, USA).

### 2.2. Cell Toxicity Assay

U87eGFP cells were plated in a 96-well plate at a density of 5000 cells per well in the culture medium. After 24 h, cells were treated with various concentrations of the compounds and were incubated for 72 h at 37 °C in an atmosphere of 5% CO_2_. The assays were performed in triplicates. Cell Counting Kit-8 assay from Bimake, (Houston, TX, USA) was used to assess the cell toxicity. The media was aspirated, and 200 µL of 10% CCK-8 solution in the complete growth medium was added. Absorbance was measured at 450 nm using an Infinite 200 Pro plate reader (Tecan, Männedorf, Switzerland) after 1.5 h of incubation at 37 °C. The viability of cells in the treated group was expressed after normalizing to the control group. Using Prism software (GraphPad, Boston, MA, USA), graphs were plotted, and IC_50_ was determined. U251 and H4 cells were treated with CA to assess the impact on viability, similar to the viability assessment assay performed for U87eGFP. Three independent experiments were performed for each of the cell lines.

### 2.3. Clonogenic Assay

U87eGFP cells were plated in 12-well plates at 62,000 cells per well and after 24 h cells were either left untreated or treated in triplicate for 72 h with CA, TCA and MCA at 40 µM (IC_30_ concentration). After this, cells were trypsinized and replated in 6-well plates at a density of 500 cells per well and incubated in media without the compounds for 14 days. Colonies were fixed and stained with crystal violet solution. For quantitative estimation of the colony forming efficiency, crystal-violet-stained colonies were lysed in 1% SDS, and the absorbance of the resulting solution was measured at 540 nm. Values of the treated groups were normalized to the control group and represented as percent of control, as described previously [9].

### 2.4. Flow Cytometry Assays

U87eGFP cells were plated in 12-well plates at 62,000 cells per well and after 24 h, cells were either left untreated (control) or treated with either CA, TCA or MCA at the IC_50_ concentration of 80 µM. Experiments were performed in triplicate, and cells were incubated for 72 h. After 72 h of incubation, cells from the media were collected, followed by procuring adhered cells via trypsinization. Cells from triplicate treatments were pooled and proceeded with Luminex Muse flow cytometry assays [10,11]. The following assay kits were used: Oxidative Stress kit (MCH100111), Annexin V and dead cell kit (MCH100105), MultiCaspase kit (MCH100109) and Mitopotential kit (MCH100110) as per the manufacturers’ instructions (Luminex Corporation, Austin, TX, USA).

### 2.5. Proteomic Analysis

Proteomic analysis was performed via 2D DIGE and mass spectrometry, which was conducted by Applied Biomics Inc. (Hayward, CA, USA) employing previously published methodologies [10,11]. U87eGFP cells were treated with 40 µM and 80 µM concentrations of CA. Control cultures (no treatment) were maintained in parallel. Cells from control samples and treated samples were collected, washed with 1× PBS and then stored at −80 °C prior to sending the samples to Applied Biomics, Inc. (Hayward, CA, USA) on dry ice for proteomic analysis. Protocol was performed as described previously [12,13].

### 2.6. Statistical Analysis

Two-way ANOVA with Dunnett’s multiple comparison test and alpha set to 0.05 and Ordinary one-way ANOVA with Dunnett’s multiple comparison test were calculated and mentioned in the legend section for figures.

## 3. Results

### 3.1. Impact of CA, TCA and MCA on U87eGFP Cells

**Figure 1:**
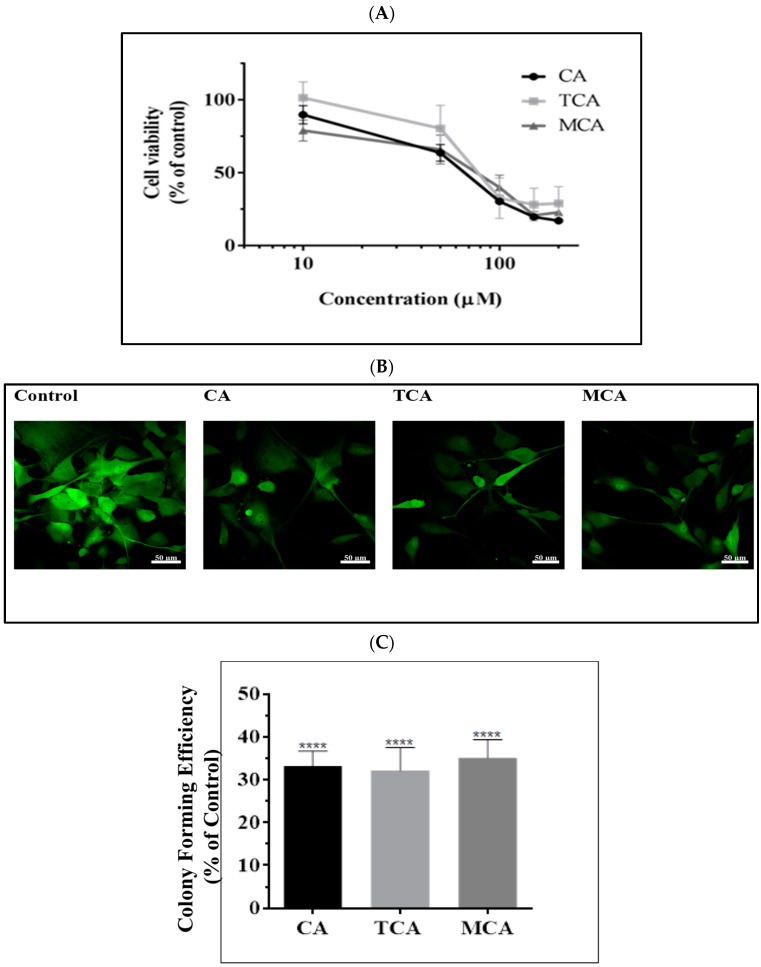
Inhibition of viability of U87eGFP cells with CA, TCA and MCA. (**A**) U87eGFP cells were plated in 96 well-plates at a density of 5000 cells/well. After 24 h, the cells were treated with various concentrations of CA, TCA and MCA ranging from 10 µM to 200 µM for 72 h. CCK-8 assay was performed to assess the cell viability. The data points are the mean of three replicates, and three such independent experiments were performed. The percentage of viable cells in treatment groups was calculated by considering untreated control values as 100%. There was a significant decrease in cell viability for all treatment groups. For both the CA- and MCA-treated group, the *p*-value < 0.0001 from 50 µM onwards, and for the TCA-treated group, the *p*-value < 0.0001 from 100 µM onwards. (**B**) To assess whether changes in cell number had occurred earlier during the treatment period, U87eGFP cells were treated with 40 µM (IC_30_) concentration of CA, TCA and MCA and incubated for 72 h and were fixed and imaged using a Zeiss 700 confocal microscope with 20× objective with a scale bar of 50 microns. (**C**) Cells were treated for 72 h with 40 µM of either CA, TCA or MCA and were reseeded in 6-well-plates at a density of 500 cells per well and allowed to form colonies over a period of 14 days. The colonies were fixed and stained with crystal violet. For quantitative analysis, fixed colonies were lysed with 1% SDS, and an absorbance reading was taken at 540 nm. The data points are mean of three replicates, and three such independent trials were performed. The absorbance values for treatment groups were normalized to the control, and there was a significant decrease in the colony-forming efficiency of the treated cells when compared to control as per ordinary one-way ANOVA with Dunnett’s multiple comparison test, and the **** *p*-value was less than 0.0001.

We opted to investigate the effects of CA, TCA and MCA on U87eGFP cells. To assess the potential of the compounds in inhibiting the viability of these cells, they were treated with varying concentrations of the three compounds for 72 h. All three compounds exhibited inhibition of cell viability in a dose-dependent manner, as shown in Figure 1A. The inhibition of cell viability was assessed with CCK-8 assay, and the IC_50_ for CA, TCA and MCA was in the range of 70–80 µM with a *p* value < 0.05. To assess the effect on cell number during the earlier times of treatment, U87eGFP cells were treated with CA, TCA and MCA at an IC_30_ concentration of 40 µM, and confocal images were taken using a Zeiss 700 confocal microscope. As seen in Figure 1B, the number of cells were reduced when compared to the controls in all the treated samples. Further, to investigate whether there was an impact on the proliferation of treated cells at this concentration, clonogenic assay was performed at the same concentration. On normalizing to the control, the percentage of the colony-forming efficiency of each of the treated samples was significantly lower than the control Figure 1C. Furthermore, there was a sustained effect because the decrease in the number of colonies in the treated group was maintained on withdrawal of the compounds. In studies with U251 and H4 cells with CA, a dose dependent inhibition of cell viability was also observed in both the cell lines with an IC_50_ of 50–60 µM for U251 and 80–90 µM for H4 as shown in Supplementary Figure S1.

### 3.2. Reactive Oxygen Species Levels Were Elevated in U87eGFP Cells after Treatment with CA, TCA and MCA

**Figure 2:**
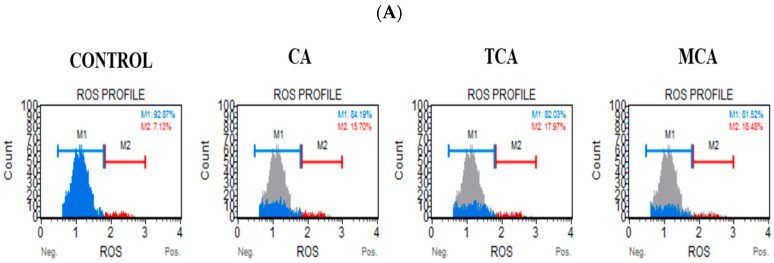

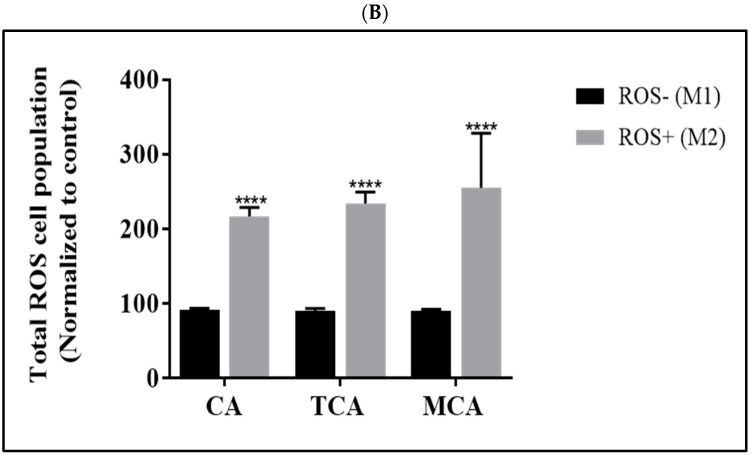
Profile of reactive oxygen species levels on treatment with CA, TCA and MCA in U87eGFP cells. (**A**) Representative ROS curves obtained for U87eGFP cells untreated or treated with 80 µM of either CA, TCA or MCA. (**B**) After normalizing to the control group, there was a significant increase in the ROS+ cell population as per 2-way ANOVA with Dunnett’s multiple comparison test and a **** *p*-value < 0.0001.

To further elucidate the mechanism by which cell viability was impacted in U87eGFP cells by CA, TCA and MCA, studies were performed to assess the production of reactive oxygen species (ROS) using an Oxidative stress kit (as described in Materials and Methods). For the experiment, the cells were treated with the IC_50_ concentration (80 μM) of each of the compounds and incubated for 72 h. An elevation in ROS was observed after treatment with each of the compounds. Representative curves obtained for the treated samples in comparison with the curves obtained for the control sample for each of the compounds are shown Figure 2A. The percentage values normalized to the control values are shown in Figure 2B. In all the treated samples, there was an increase in ROS production in the U87eGFP cells.

### 3.3. Programmed Cell-Death Pathway Was Impacted by CA, TCA and MCA in U87eGFP Cells

**Figure 3:**
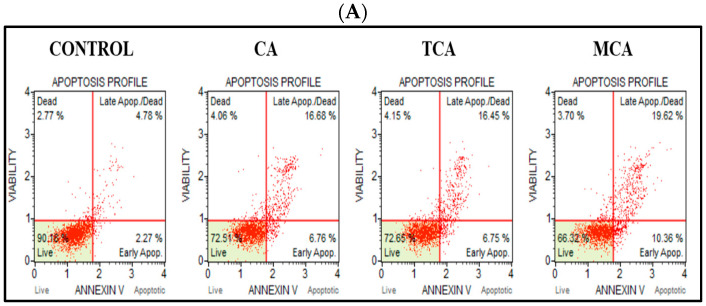

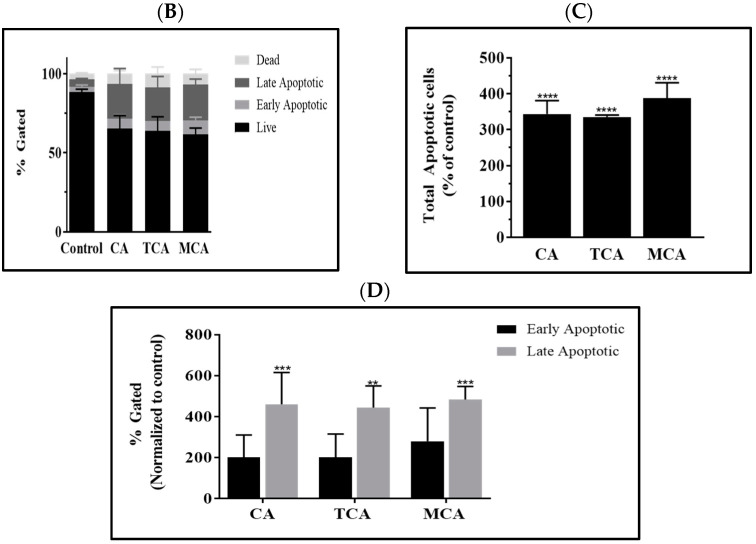
Analysis of apoptosis in U87eGFP cells treated with CA, TCA and MCA. (**A**) Representative scatter plots from Annexin V flow cytometry of U87eGFP cells treated with 80 µM of either CA, TCA or MCA for 72 h. (**B**) Percent gated profile showing the different cell population of untreated and treated cells averaged from three independent experiments. A significant change was seen in the 4 different cell populations analyzed in the treated groups as per 2-way ANOVA with Dunnett’s multiple comparison test and the *p*-value was 0.0001. (**C**) Bar graph showing total apoptotic cells normalized to the control with the mean and standard deviation from 3 trials. Total number of apoptotic cells increased significantly in treated groups as per Ordinary 1-way ANOVA with Dunnett’s multiple comparison test and a **** *p*-value < 0.0001. (**D**) After normalizing to the control group, both early and late apoptotic cell populations are represented as ** = 0.0011 and *** < 0.001 as per 2-way ANOVA with Dunnett’s multiple comparison test.

After observing inhibition of cell viability and significant impact on the colony-forming efficiency in U87eGFP cells by CA, TCA and MCA, as well as an increase in ROS levels, we proceeded further to elucidate the mechanism of action. We opted to investigate the effect of all three compounds on the programmed cell-death pathways. To study the effect on the extrinsic pathway, we performed Annexin V flow cytometric assay on treating cells at the IC_50_ concentration of 80 µM of each of the compounds for 72 h. Representative scatter plots of the control sample and samples treated with CA, TCA and MCA are shown in Figure 3A. Percent gated profiles of each of the cell populations in the untreated and treated groups are shown in Figure 3B. The total number of apoptotic cells in the treatment groups normalized to untreated cells and averaged from three independent experiments are represented by a bar graph shown in Figure 3C. The total number of apoptotic cells increased significantly in treated samples. The percent of early and late apoptotic cell populations normalized to the control population is represented in Figure 3D. A statistically significant increase in the late apoptotic population was observed in all the treated groups.

### 3.4. Multicaspase Was Elicited by CA, TCA and MCA in U87eGFP Cells

**Figure 4:**
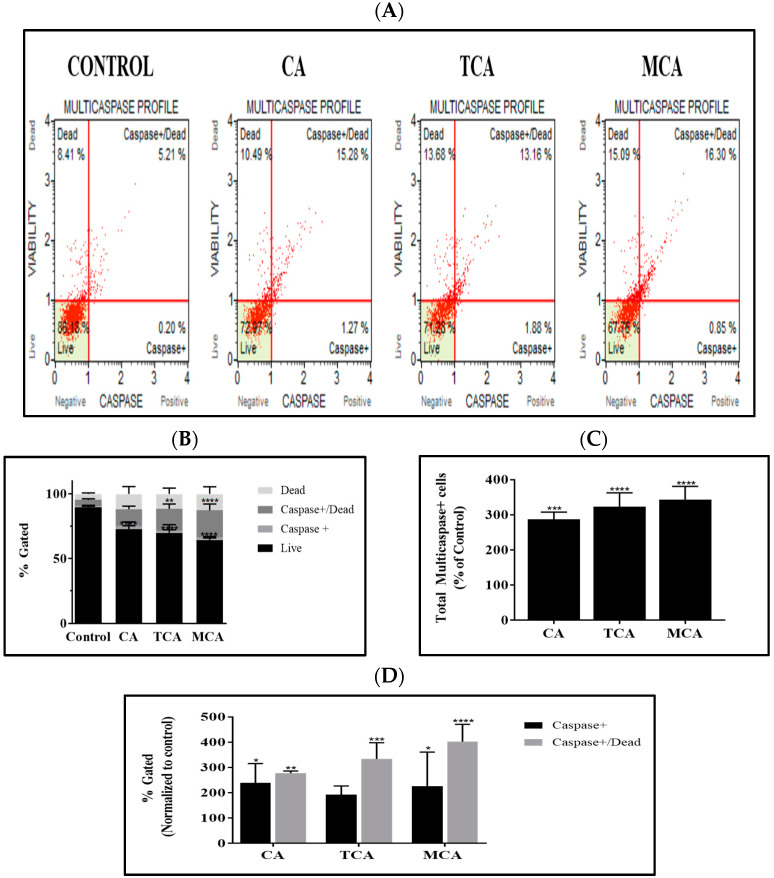
Activation of multicaspase in CA-, TCA- and MCA-treated U87eGFP cells. (**A**) Representative multicaspase scatter plots of U87eGFP cells untreated and treated with 80 µM of either CA, TCA or MCA. (**B**) Histogram showing the multicaspase profile of untreated and treated cells averaged from three independent experiments. A significant decrease in live cell population with a *p*-value < 0.0001 and a significant increase in the Caspase+/Dead population of the TCA (*p*-value = 0.0023)- and MCA (*p*-value < 0.0001)-treated groups was seen, as per 2-way ANOVA with Sidak’s multiple comparison test. (**C**) Bar graph showing total multicaspase+ cells normalized to the control with the mean and standard deviation from 3 trials. The total number of caspase+ cells increased significantly in treated groups as per Ordinary 1-way ANOVA with Dunnett’s multiple comparison test and *** = 0.0002 and **** < 0.0001. (**D**) After normalizing to the control group, both the caspase+ and caspase+/dead cell populations are represented with * = 0.0243 for CA and 0.0411 for MCA, ** = 0.0046, *** = 0.0004 and **** < 0.0001, as per 2-way ANOVA with Dunnett’s multiple comparison test.

On observing the extrinsic pathway being invoked by CA and its isomer/analog, we investigated whether caspases, in particular MultiCaspase, were elicited. MultiCaspase activation was monitored by flow cytometry using the MultiCaspase assay kit. MultiCaspase scatter plots of U87eGFP cells untreated (control) and treated with 80 µM of either CA, TCA or MCA are represented in Figure 4A. The MultiCaspase profile of untreated and treated cells averaged from three independent experiments are shown in Figure 4B. A statistically significant decrease in the live cell population and a significant increase in the Caspase+/Dead population in TCA- and MCA-treated groups was observed. A bar graph showing total multicaspase+ cells normalized to the control with the mean and standard deviation from 3 trials is shown in Figure 4C. There was a statistically significant increase in the total number of caspase+ cells in the treated groups. After normalizing to the control group, both the caspase+ and caspase+/dead cell populations increased significantly, as shown in Figure 4D.

### 3.5. The Intrinsic Programmed Cell-Death Pathway Was Impacted by TCA and MCA in U87eGFP Cells

**Figure 5:**
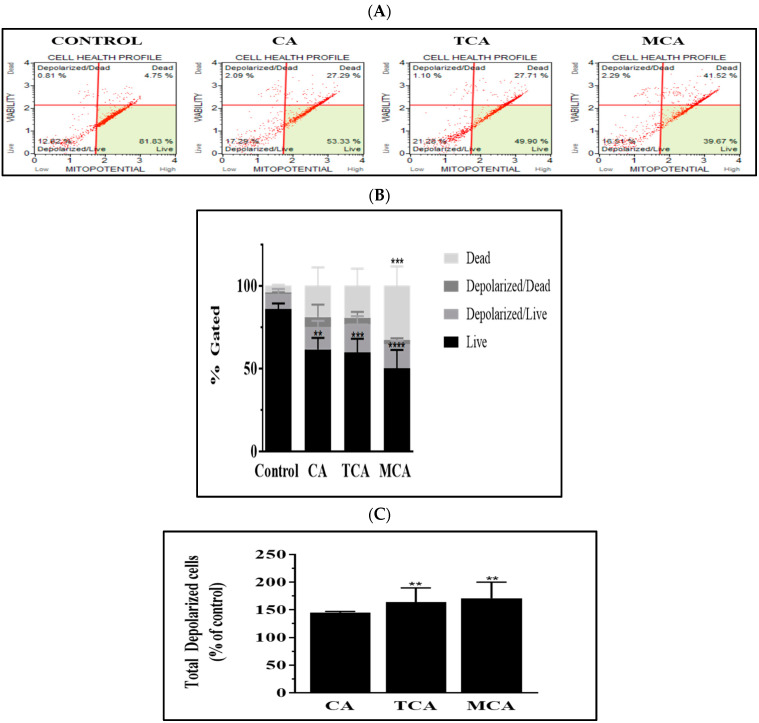
Mitochondrial depolarization in U87eGFP cells treated with TCA and MCA. (**A**) Representative scatter plots from flow cytometry using a MitoPotential kit to assess the depolarization of the mitochondrial membrane potential in U87eGFP cells treated with 80 µM either of CA, TCA or MCA for 72 h. (**B**) Histogram (% gated) showing the profile of cell population of untreated (control) and treated cells averaged from three independent experiments. A significant decrease in the live population of all the treated groups was seen as per 2-way ANOVA with Sidak’s multiple comparison test and a *p*-value of 0.0014 for CA, *** = 0.0005 for TCA and **** < 0.0001 for MCA. (**C**) Bar graph showing total depolarized cells normalized to the control with the mean and standard deviation from 3 trials. Total number of depolarized cells increased significantly in TCA- and MCA-treated groups as per Ordinary 1-way ANOVA with Dunnett’s multiple comparison test and ** *p*-values of 0.0093 and 0.0052, respectively.

A significant impact was observed on the extrinsic programmed cell-death pathway by CA, TCA and MCA in U87eGFP cells, which led us to investigate whether the three compounds had an impact on the intrinsic programmed cell-death pathway as well. To assess the possible effect on the mitochondria, we performed flow cytometry using a mitopotential depolarization analysis kit. Representative scatter plots of U87eGFP cells treated with 80 µM of either CA, TCA or MCA after 72 h of incubation are shown in Figure 5A. The percent gated profiles of all the populations of cells of untreated and treated cells averaged from three independent experiments are shown in Figure 5B. A significant decrease in the live population of cells in all the treated groups was observed. Total depolarized cells normalized to the control with the mean and standard deviation from three trials are represented by a bar graph in Figure 5C. There was a statistically significant increase in the total number of depolarized cells in the TCA- and MCA-treated groups.

### 3.6. Proteomic Analysis Reveals Entities of Pivotal Signaling Pathways Differentially Regulated in U87eGFP Cells after Administration of CA

**Figure 6:**
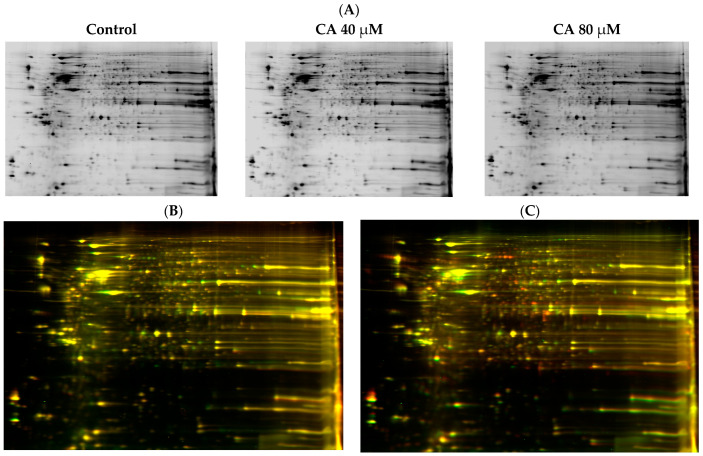

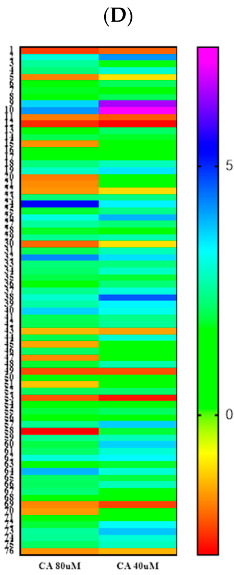
Proteomic analysis of U87eGFP cells after treatment with CA employing 2D DIGE. Control U87eGFP cells as well as CA-treated samples, 40 µM and 80 µM samples were processed to extract proteins as described in ‘Materials and Methods’. A clear separation of proteins in each of the samples was observed, as shown in (**A**). Cell lysates of the control group were combined with Cy2 dye, the 40 µM treated sample was tagged with Cy3 and the 80 µM treated sample was tagged with Cy5. The overlay of the gel of CA-40 µM-treated sample and control sample is shown in (**B**) along with the overlay of the gel of CA-80 µM-treated sample and control sample in (**C**). A heatmap showing the fold change in expression of the proteins in the 40 µM- and 80 µM-treatment groups is shown in (**D**).

To further delineate the key entities that could have been impacted, protein profiling of U87eGFP cells treated with CA 40 µM and 80 µM along with untreated cells was performed using 2D gel electrophoresis and mass spectrometric analysis (as described in ‘Materials and Methods’). Clear separation of proteins from each of the cell extracts is shown in Figure 6A. For proteomic analysis, the control proteins were tagged with Cy2, the 40 µM treated sample was tagged with Cy3 and the 80 µM treated sample was tagged with Cy5 prior to being subjected to electrophoresis. An overlay of the gel of CA treated at 40 µM and the control sample gel are shown in Figure 6B. Similarly, overlay of the gel of CA treated at 80 µM and the control sample gel are shown in Figure 6C. The number of differentially expressed proteins is shown in the heatmap of proteins in Figure 6D.

Based on the fold changes obtained, we selected upregulated and downregulated proteins for further proteomic analysis via mass spectrometry. The details of the molecules profiled are represented in Table 1. Among the upregulated proteins, the entities belonged to pivotal signaling pathways. Actin cytoplasmic 2 which plays a role in ECM-circuit and 60 S ribosomal protein L17 which plays a role in regulating cell proliferation in certain tissues were also upregulated. Among components that control the cell cycle, thymidylate synthase was upregulated. Furthermore, stress protein such as biliverdin reductase A was also upregulated.

Among the downregulated proteins, certain pivotal components of key signaling pathways were impacted. Importantly, phosphomevalonate kinase, which plays a role in the proliferation of cells as well as in the immune pathway and pyruvate kinase (PKM2) of the glycolytic pathway, which is central to the Warburg effect, was downregulated. Thus, cinnamaldehyde treatment had a profound impact on the viability of U87eGFP glioblastoma cells.

**Figure 7:**
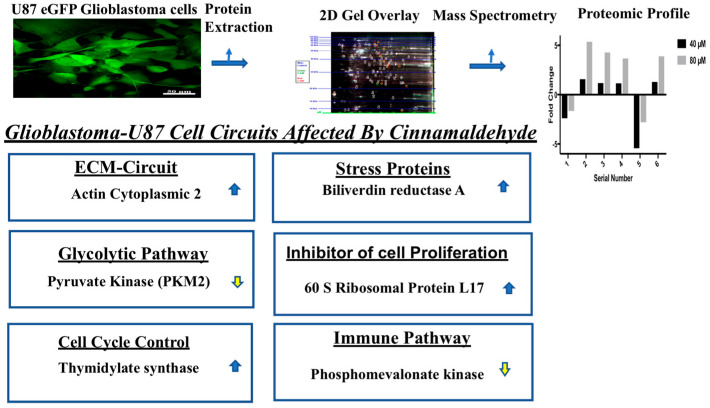
Cellular pathways impacted by cinnamaldehyde. Upregulated (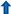) and downregulated (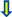) entities in glioblastoma cells after treatment with cinnamaldehyde.

The details of the cell signaling pathways and the entities impacted by CA are represented in the schematic diagram in Figure 7.

## 4. Discussion

The need for efficacious drugs for treating aggressive brain tumors such as glioblastoma multiforme that could have potentially fewer side effects led us to investigate the effect of a purified, single entity of a naturally occurring compound from cinnamon such as cinnamaldehyde (CA). CA has been reported to inhibit proliferation of cancer cells of varied origins [14]. In the present study, the viability and proliferation potential of U87eGFP cells were impacted by CA. Furthermore, in the clonogenic assay, not only was there an impact observed on the proliferation potential of U87eGFP cells, but also a sustained response was observed on withdrawal of the compounds. Notably, an increase in ROS was observed in all the treated samples. Impact on the ROS levels is reported as one of the modes of action of many anticancer agents [15]. Furthermore, we observed cell death of U87eGFP cells occurring through the extrinsic programmed cell-death pathway, resulting in a significant increase in the population of apoptotic cells as well as cells with activated multicaspase. Additionally, the intrinsic cell-death pathway was impacted, leading to depolarization of the mitochondrial membrane potential; the increased population of cells with depolarized mitochondrial membranes was statistically significant in the TCA- and MCA-treated groups. With respect to the mechanism of inhibition in U251 and H4 cell lines by CA, we envision that it could be different from that is operational in U87 cells. U251 glioblastoma and H4 neuroglioma have different genetic makeups compared to U87. Therefore, analyses of both extrinsic and intrinsic pathways need to be investigated to arrive at conclusions on the underlying mechanism(s) for inhibition of proliferation in these cell lines.

To further delineate the entities impacted by CA in U87eGFP cells, protein profiling using techniques such as 2D gel electrophoresis and mass spectrometry were performed, which are more sensitive and can detect protein changes in the femtomolar range. The technologies employed in the present study are more sensitive than Western blots and ELISA, which are semiquantitative [16]. Protein-profiling data revealed key upregulated and downregulated entities encompassing various pivotal cell signaling pathways.

Among the upregulated entities was actin cytoplasmic 2. Cytoplasmic actin plays an important role in the cell and in cancers. With respect to cell transformation, actin cytoskeletal protein could play a structural and functional role in the cell’s extracellular matrix (ECM) and is also involved in the communication between the nucleus and ECM [17]. Further, it is reported that a combination of FDA-approved oncology drugs with effects on the cytoskeleton could be considered as a combination therapy for GBM in future studies [18]. Thus, a combination therapy of drugs that act on the cytoskeleton with CA could be considered as a novel therapeutic option for GBM treatment if resistance occurs.

The ribosomal protein, identified as 60 S ribosomal protein L17, was also among the upregulated proteins by CA. Interestingly, ribosomal proteins (RPs) have been reported to have extra-ribosomal functions [19] and the ribosomal protein L17 acts as a negative regulator of cell proliferation and inhibits vascular smooth muscle (VSM) growth [20]. We envision that an increase in this ribosomal protein could impact cell proliferation of glioblastoma cells, especially considering the propensity of GBM for neovascularization.

Another upregulated entity was biliverdin reductase A (a cytoprotective agent). Increase in this molecule indicates that the treated cells are under stress and are activating molecules in the survival pathway. Interestingly, biliverdin reductase-based peptides could be considered as a novel therapeutic option because they inhibit cell proliferation [21]. Therefore, biliverdin reductase A peptides as inhibitors could be considered in combination with CA to prevent the cancer cells from using this pathway to become resistant. Another molecule that was upregulated was thymidylate synthase. Thymidylate synthase is under the control of CDK4 [22]. Therefore, CDK4 inhibitors, which are already under clinical trials, could be used in combination therapy to prevent the cancer cells from escaping cell death if resistance occurs.

Among the pathways controlling immunity, the mevalonate pathway is shown to control chemoresistance, and impacting this pathway could cause immunological cell death [23]. Moreover, this pathway is involved in coordinating the input of energy requirements and the proliferation of cells [24]. A decrease in phosphomevalonate kinase observed during CA treatment of U87eGFP cells in the present study could have a significant effect on proliferation. In fact, we observed an impact caused by CA on cell proliferation in our cellular level studies. Furthermore, the decrease in phosphomevalonate kinase could be harnessed for alerting the immune system to the pathways that guard the cancers from being attacked because the mevalonate pathway is pivotal to cancer immune surveillance [25]. Of noteworthy is the report that patient-derived brain tumor initiating cells (BTIC) from glioblastoma are maintained by a Myc-regulated mevalonate pathway [26]. Similarly, the lipid-lowering drug Lovastatin is reported to possibly have an effect on glioblastoma stem cells by interfering with the mevalonate pathway [27]. Therefore, we envision that a reduction in phosphomevalonate kinase in the present study with CA could have an inhibitory effect on the maintenance of BTIC in glioblastomas, thus possibly preventing recurrence of this devastating cancer and providing a beneficial therapeutic effect. Notably, the key oncoprotein Myc has proven to be a difficult target to be inhibited and is considered undruggable at present. Thus, to circumvent the problem, impacting pathways controlled by Myc such as the mevalonate pathway by decrease in phosphomevalonate kinase could be considered as one of the strategies to inhibit its role in cancer-cell proliferation.

One of the hallmarks of cancer cells is metabolic reprogramming, wherein aerobic glycolysis occurs instead of the regular respiratory pathway to harness energy, leading to what is termed the ‘Warburg effect’ [28]. One of the proteins that plays a key role in this metabolic reprogramming is pyruvate kinase M2 (PKM2), which is upregulated in many tumors including glioblastomas [29,30,31]. Further, microRNA-326 has been reported to regulate PKM2 and impact glioma cell survival [32]. Furthermore, it has also been reported that in U87MG, inhibition of PKM2 led to late apoptotic cell population [31]. In fact, in the present molecular mode of action study, we observed an increase in the late apoptotic cell population in CA-treated cultures.

The drug TMZ, which is part of the current standard of care for glioblastomas, causes changes in cell metabolism and, in particular, decreased expression and activity of pyruvate kinase-PKM2. The significant decrease in the conversion of pyruvate to lactate observed in TMZ-treated glioblastoma cells is monitored by administering hyperpolarized (1–13 C) pyruvate and tracked by employing magnetic resonance spectroscopic imaging (MRSI). This method has been shown to be more reliable, more rapid and forms a biosensor for assessing the therapeutic effect of TMZ over measuring the tumor size, which takes days [33]. In the present study with CA, one of the proteins that was downregulated was PKM2, which was detected through protein profiling. Therefore, hyperpolarized pyruvate (1–13 C pyruvate) used in the assessment of therapeutic outcomes of TMZ in glioma cells could be employed to assess the effect of CA as well. Importantly, preferential expression of PKM2 has been reported in glioblastoma cells, and minimal expression has been observed in normal brain cells. Employing positron emission tomography (PET) tracer [^18^F]DASA-23, which comprises 1-((2-fluoro-6-[^18^F] fluorophenyl)sulfonyl)-4((methoxyphenyl)sulfonyl)piperazine, visualization of aberrant expression of PKM2 in cell culture, mouse models of glioblastoma multiforme (GBM), healthy human volunteers and patients with GBM have been reported [34]. Therefore, the same technology could be employed to assess the effect of CA in GBM as it affects the protein levels of PKM2. Future studies with CA, which has multifaceted effect as a monotherapy, and also studies in combination with current chemotherapeutics are warranted to provide the unmet need of efficacious treatment for aggressive brain tumors such as the glioblastoma multiforme.

## Figures and Tables

**Table 1:** Differentially Regulated Entities using Cinnamaldehyde in U87eGFP Glioblastoma Cells.

Fold Change		Protein ID
40 µM	80 µM	
−2.4	−1.66	Pyruvate Kinase 2 (PKM2)
+1.56	+5.36	Actin cytoplasmic 2
+1.18	+4.27	Biliverdin reductase A
+1.15	+3.67	Thymidylate synthase
−5.44	−2.81	Phosphomevalonate kinase
+1.29	+3.89	60 S ribosomal protein L17

+ values represent upregulated and − values are downregulated entities.

## Data Availability

All the data relating to this article are presented in the manuscript.

## Supplementary Materials

The following supporting information can be downloaded at https://www.mdpi.com/article/10.3390/cells12091277/s1: Figure S1: Impact of CA on U251 glioblastoma cells and H4 neuroglioma cells.
