# Supplementary material for: Disruption of Glioblastoma Multiforme Cell Circuits with Cinnamaldehyde Highlights Potential Targets with Implications for Novel Therapeutic Strategies
Source: Cells. 2023 Apr 28;12(9):1277. doi: 10.3390/cells12091277 (PMC10177046; doi:10.3390/cells12091277)
Supplement: Supplementary file 1 [file cells-12-01277-s001.zip › cells-2306467-supplementary.pdf]

**Supplementary Figure**

**Supplementary Figure 1.** Impact of CA on U251 glioblastoma cells and H4 neuroglioma cells. On observing significant impact on U87eGFP cells by CA and its isomers, we proceeded to investigate whether CA impacted other brain tumor /glioma cells like U251 and H4. For this analysis, U251 and H4 cells were treated with varying concentrations of CA and incubated for 72 h. A dose dependent inhibition of cell viability was observed in both the cell lines with an $IC_{50}$ of 50–60 μM for U251 and 80–90 μM for H4 as shown in supplementary Figure 1(A), and (B).

(**A**)

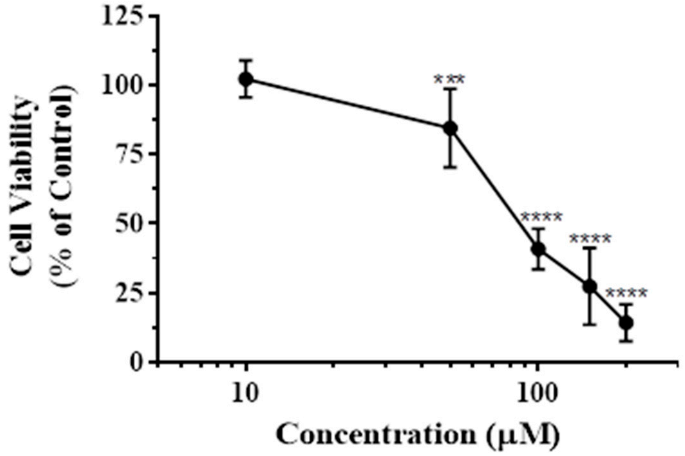


(**B**)

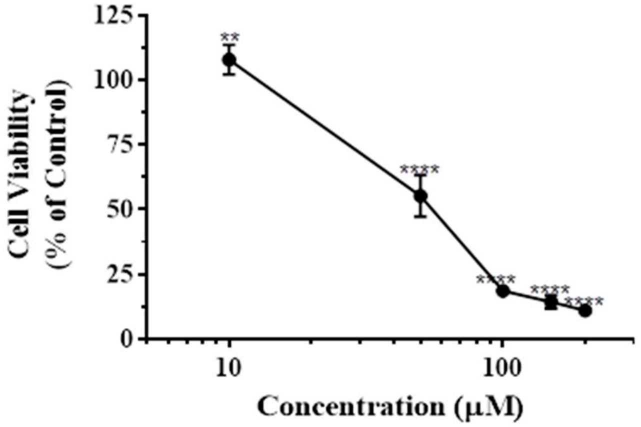


**Supplementary Figure S1.** Inhibition of viability of U251 and H4 cells by CA. (A) U251 and (B) H4 cells were plated in 96 well-plates at a density of 5000 cells/well. After 24 h, the cells were treated with various concentrations of CA ranging from 10 μM to 200 μM for 72 h. CCK-8 assay was performed to assess the cell viability. The data points are mean of three replicates and three such independent experiments were performed. Percent of viable cells in treatment groups was calculated by considering untreated control values as 100% and a significant decrease in cell viability was observed in U251 and H4 cells. *p*-values: ** =0.0037, *** = 0.0003,****< 0.0001.
